# Effect of ethnicity on HbA1c levels in individuals without diabetes: Systematic review and meta-analysis

**DOI:** 10.1371/journal.pone.0171315

**Published:** 2017-02-13

**Authors:** Gabriela Cavagnolli, Ana Laura Pimentel, Priscila Aparecida Correa Freitas, Jorge Luiz Gross, Joíza Lins Camargo

**Affiliations:** 1 Graduate Program in Endocrinology, Universidade Federal do Rio Grande do Sul (UFRGS), Porto Alegre, Brazil; 2 Centro Universitário FSG, Caxias do Sul, Brazil; 3 Laboratory of Transplantation Immunology, Santa Casa de Misericordia de Porto Alegre, Porto Alegre, Brazil; 4 Endocrinology Division, Hospital de Clínicas de Porto Alegre, Porto Alegre, Brazil; University of Oxford, UNITED KINGDOM

## Abstract

**Aims/Hypothesis:**

Disparities in HbA1c levels have been observed among ethnic groups. Most studies were performed in patients with diabetes mellitus (DM), which may interfere with results due to the high variability of glucose levels. We conducted a systematic review and meta-analysis to investigate the effect of ethnicity on HbA1c levels in individuals without DM.

**Methods:**

This is a systematic review with meta-analysis. We searched MEDLINE and EMBASE up to September 2016. Studies published after 1996, performed in adults without DM, reporting HbA1c results measured by certified/standardized methods were included. A random effects model was used and the effect size was presented as weighted HbA1c mean difference (95% CI) between different ethnicities as compared to White ethnicity.

**Results:**

Twelve studies met the inclusion criteria, totalling data from 49,238 individuals. There were significant differences between HbA1c levels in Blacks [0.26% (2.8 mmol/mol); 95% CI 0.18 to 0.33 (2.0 to 3.6), p <0.001; I^2^ = 90%, p <0.001], Asians [0.24% (2.6 mmol/mol); 95% CI 0.16 to 0.33 (1.7 to 3.6), p <0.001; I^2^ = 80%, p = 0.0006] and Latinos [0.08% (0.9 mmol/mol); IC 95% 0.06 to 0.10 (0.7 to 1.1); p <0.001; I^2^ = 0%; p = 0.72] when compared to Whites.

**Conclusions/Interpretation:**

This meta-analysis shows that, in individuals without DM, HbA1c values are higher in Blacks, Asians, and Latinos when compared to White persons. Although small, these differences might have impact on the use of a sole HbA1c point to diagnose DM in all ethnic populations.

## Introduction

HbA1c has been considered the reference test for the assessment of glycaemic control in individuals with diabetes mellitus (DM) for over three decades [1–3]. Also, HbA1c levels are important for therapeutic adjustment and to predict the risk of developing chronic diabetic complications [1]. An absolute increase of 1% in HbA1c value is associated with 15–20% elevations in the cardiovascular risk [4] and absolute reductions of 1 to 2% are related to significant decreases of risk for microvascular complications [2, 3]. Since 2010, this test has also been used as a diagnostic criterion with the cut-off point of 6.5% (48 mmol/mol) recommended in confirming the presence of DM [1, 5]. This cut-off has high specificity, but low sensitivity, and its isolated use for the diagnosis of DM has been questioned [6, 7].

Although HbA1c has advantages over the traditional tests used to diagnose DM–fasting plasma glucose (FPG) and oral glucose tolerance test (OGTT)–and despite the international efforts to harmonize and standardize laboratory methods, there are some pathophysiological and methodological situations that may affect the interpretation of HbA1c test and, in specific cases, limit its use [8, 9]. In a recent meta-analysis we have shown that uraemia, HbS and HbC do not seem to be associated with alterations in HbA1c results in individuals without DM [10]. In an additional study, we showed that iron deficiency anaemia affects HbA1c results by upward changes and that this effect depends on the degree of anaemia [11].

In addition to these pathological interferences, studies have shown differences in HbA1c levels between individuals with DM from different ethnicities that do not seem to be associated with glucose changes or haematological disorders [12, 13]. These ethnic disparities in HbA1c levels have been recognized for many years, but have generally been attributed to differences in the access to health care for different ethnic groups and populations [13]. Most studies that compared HbA1c levels among ethnicities were performed in individuals with impaired glucose tolerance or overt DM, consequently, the results had to be adjusted for glucose levels [14–16]. There are still controversial results regarding the differences in the long-term complications and mortality rates [14, 17–25].

A few data evaluating differences in HbA1c values among individuals without DM from different ethnic groups, without glucose variability and treatment effects, were reported [18, 20, 22, 25, 26]. Under this condition, the constant blood glucose levels minimize the HbA1c variability. Thus, in order to assess the presence and the degree of these ethnic differences, we performed a systematic review and meta-analysis of studies that compared the effect of ethnicity on the HbA1c levels in individuals without DM.

## Methods

This systematic review was performed in accordance with the Cochrane Collaboration [27] and reported in agreement with the Meta-analysis of Observational Studies in Epidemiology (MOOSE) [28].

### Data sources and searches

The search was conducted to select studies that evaluated the HbA1c levels in different ethnic groups in the absence of DM. The databases used in the search were MEDLINE and EMBASE through to September 2016. The following keywords were used: "glycated haemoglobin" and "ethnicity". The complete search strategy is described in S1 Appendix. All possible qualified studies were considered for review, regardless of the language. We also performed a manual search, using reference lists of all articles included and relevant reviews in order to find other possibly relevant studies and when necessary, we contacted authors to request information necessary to include studies.

### Study selection

Studies were eligible for inclusion if they had been performed in adults above 18 years without DM [1, 5], to exclude the possible variability of HbA1c due to glucose fluctuations. We, therefore, considered that the variability in HbA1c levels was only due to the differences in ethnic groups. Necessarily, the articles should have a group with White persons and a group of a different ethnicity. Articles that included individuals with DM were also included in our systematic review if they had at least one group without DM and HbA1c levels for different ethnic groups to perform comparisons. Articles that included only pre-DM individuals were excluded.

The methods for HbA1c determination had to be standardized/certified by the National Glycohemoglobin Standardization Program (NGSP) and/or International Federation for Clinical Chemistry (IFCC) ([29, 30]; http://www.ngsp.org). When this information was incomplete or unavailable, we contacted the authors for confirmation of the methodology. Only articles published after 1996 were eligible, since it was from that date on that the standardization for the HbA1c methods by NGSP program started [30].

Cross-sectional, cohort and control-case studies were included if they satisfied the above criteria. Review articles, reports/case studies, animal studies, letters, abstracts without sufficient data for analysis and studies with pregnant women were excluded.

### Data extraction and quality assessment

The list of titles and abstracts resulting from the search strategy was evaluated by two independent investigators (GC and ALP). Studies that met the eligibility criteria were selected to be read in their entirety. The same reviewers performed the data extraction independently. Disagreements were resolved by consensus and, when necessary, by a third investigator (JLC).

The primary outcome required was mean and SD of HbA1C values in different ethnic groups. We used a standardized form to extract the data that included the following items: study design, diagnostic criteria for DM, total number of participants, number of participants within ethnic groups, percentage of men; mean and/or range of ages, mean and SD or SE of glycaemia and HbA1c levels and the methodology used to measure HbA1c. If mean and SD of HbA1c results were not available in the articles, we contacted the authors by email to obtain these data. When the manuscript presented sufficient data to calculate these variables, we calculated them with the following formulae: 95% CI = mean ± 1.96SE and SE = SD√n. The articles whose authors did not respond and had insufficient data for the calculations were excluded. HbA1c values are expressed in % and in mmol/mol throughout the paper, however we performed the meta-analysis with HbA1c expressed in % (http://www.ngsp.org/convert1.asp). Unadjusted data were preferable for extraction, otherwise adjusted data were considered and the adjustments were considered in the sensitivity analysis to investigate heterogeneity whenever possible. The methodological quality of each study included in the meta-analyses was also assessed independently by two reviewers (GC and ALP) through a questionnaire developed by the authors. The questionnaire was adapted from the Newcastle–Ottawa scale and the Laboratory Medicine Best Practices Initiative Guide to Rating Study Quality [31, 32], and the questions were related to the study population (age, gender and clinical origin), selection of participants (diabetes diagnostic tests), study design, classification of ethnic groups and statistical analysis. The overall quality of each study was arbitrarily classified as: 8–10 stars = good; 5–7 stars = moderate and less than 5 stars = poor (S2 Appendix).

### Data synthesis and analysis

Meta-analyses were performed using a random effects model and the effect size was presented as weighted HbA1c mean difference (95% CI). For data analysis, the White ethnic group was considered the control group. We made this choice taking into consideration that most studies found in the literature have compared the White ethnicity with other ethnicities.

The heterogeneity among the studies was evaluated by the inconsistency test (I^2^), where 0%, 25%, 50% and 75% indicate, respectively, absent, low, moderate and high heterogeneity among studies [28]. We explored the heterogeneity by re-running the meta-analysis removing studies one at a time to determine the contribution of each study to heterogeneity.

Meta-analyses were performed using Review Manager Version 5.3 (Revman—Cochrane Collaboration). Funnel plots were constructed using Stata version 10.1 software (Stata, College Station, TX). Asymmetry was assessed by visual inspection or by Egger's test if in the meta-analysis had been included at least 10 studies, as recommended, for investigation of publication bias [27,33]. A *P* value <0.05 was considered statistically significant in all analyses except for publication bias (Egger test), where a value of *P* <0.1 was considered statistically significant.

Ethics approval was not required, as we pooled previously published studies.

## Results

### Search results

**Fig 1:**
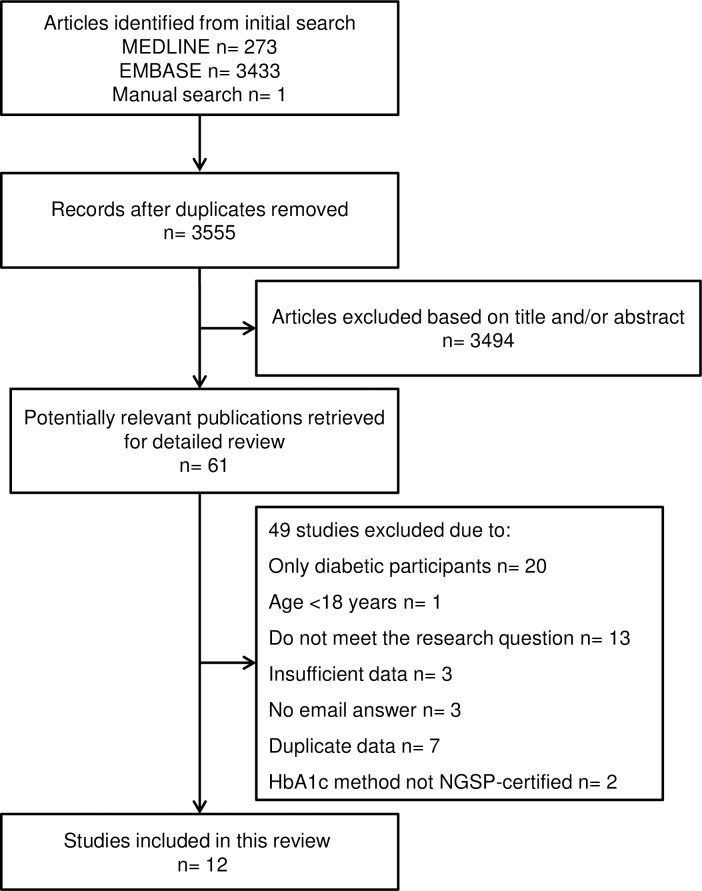
Flowchart of the article selection process.

Following the initial strategy, we identified 3,555 studies. After the examination of titles and abstracts, 61 studies were selected for a full text analysis and 12 studies met the inclusion criteria [19, 20, 22, 24, 25, 34–40]. All manuscripts have provided sufficient data for quantitative analysis, totalizing data from 49,238 individuals. Three of them were cohort studies and 9 presented cross-sectional design (Fig 1). All selected studies were published between 1999 and 2016. The characteristics and the main findings of these studies are shown in Table 1.

**Table 1:** Characteristics of 10 studies included in this review.

Study, Year, Ref Number	N; % men; Age (years)	Study Design	Population	HbA1c Method	Main Findings
**Burden, 1999 [34]**		Cross-Sectional	Healthy individuals from outside Leicester, England. Information on age, sex, ethnicity and presence of DM were obtained by questionnaire.	Immunoassay DCA 2000; Bayer Diagnostic	Indo-Asians presented higher HbA1c values than Whites [5.7% (39 mmol/mol) and 5.3% (34 mmol/mol), respectively]. Random whole blood glucose levels were higher in Indo-Asian than in White patients.
Whites	135; 45.0;NI				
Indo-Asians	127; 48.0; NI				
**Likhari, 2010 [35]**		Cross-Sectional	Individuals referred from primary care to perform OGTT at New Cross Hospital, Wolverhampton, England. Only normoglycemic individuals were included (FPG <110 mg/dL and 2hG <160 mg/dL). Ethnicity classification was by self-report.	Ion-Exchange HPLC A1c Tosoh; Tosoh Corporation	South-Asian individuals had higher HbA1c values than Whites [6.1% (43 mmol/mol) and 5.9% (41 mmol/mol), respectively] for similar FPG and 2hG levels.
Whites	103; 52.4; 63.2				
South-Asians	36; 44.4; 51.6				
**Kehl, 2011 [22]**		Cohort	Data from NHANES III 1988–1994. American individuals aged >20 years. Ethnicity and DM classification were by self-report.	Ion-Exchange HPLC Diamat Analyzer System; Bio-Rad Laboratories	HbA1c levels were higher in non-Hispanic Blacks than in Mexican-Americans and Whites [5.4% (36 mmol/mol), 5.3% (34 mmol/mol) and 5.2% (33 mmol/mol), respectively]. After adjustment for sex and age, only in non-Hispanic Whites the highest HbA1c levels were associated with overall mortality and cardiovascular disease.
Non-Hispanic Whites	5,573; 48.6; 45.9				
Non-Hispanic Blacks	3,584; 45.2; 40.0				
Mexican-Americans	3,541; 54.5; 36.3				
**Selvin, 2013 [24]**		Cross-Sectional	Participants from the ARIC study. History of DM was determined by the records at visit 2 or visit 1.	Ion-Exchange HPLC Tosoh HbA1c 2.2; Tosoh A1C G7 Tosoh Corporation	Blacks had higher HbA1c values than Whites [5.8% (40 mmol/mol) and 5.4% (36 mmol/mol), p<0.001, respectively]. There were no differences in the association of HbA1c with kidney disease and vascular outcomes in blacks compared with whites.
Whites	8,593; 44.3; 56.9				
Blacks	2,484; 35.5; 55.8				
**Chapp-Jumbo, 2012 [19]**		Cohort	Participants from the Pathobiology of Prediabetes in a Biracial Cohort (POP-ABC) study. Normoglycemic individuals (FPG <100 mg/dL and 2hG <140 mg/dL) from Memphis region, United States. Ethnicity was classified by self-report.	Ion-Exchange HPLC Bio-Rad; Bio-Rad Laboratories	HbA1c was higher in African American participants than in Whites [5.7% (39 mmol/mol) and 5.5% (37 mmol/mol), respectively] even after adjusting for age, adiposity, blood glucose, and other variables. Blacks were younger and had lower FPG, 2hG and total hemoglobin levels than Whites.
Whites	135; 31.1; 47.2				
African Blacks	167; 26.9; 43.8				
**Mostafa, 2012 [20]**		Cross-Sectional	Primary care participants of the ADDITION study. DM classification was by OGTT according to the WHO 1999 criteria.	Ion-Exchange HPLC Variant II; Bio-Rad Laboratories	HbA1c, FPG and 2hG levels were independently higher in South Asians than in Whites. The authors attributed the difference partly to factors related to glycaemia. The results adjusted for multiple regression analysis in model 2 were used in this meta-analysis.
White Europeans	4,688; NI; NI				
South Asians	1,352; NI; NI				
**Bower, 2013 [25]**		Cross-Sectional	Participants of the study NHANES 2005–2008. History of DM was defined by self-report or by insulin use.	Ion-Exchange HPLC Tosoh A1C G8; Tosoh Corporation	In people without DM both non-Hispanic blacks and Hispanics was observed higher HbA1c values compared with non-Hispanic whites [5.7% (39 mmol/mol), 5.6% (38 mmol/mol) and 5.5% (37 mmol/mol), respectively]. There were no ethnic differences in the association of HbA1c with retinopathy.
Non-Hispanic Whites	2,612; 47.8; 56.7				
Non-Hispanic Blacks	805; 45.3; 53.5				
Hispanic Americans	996; 49.2; 51.9				
**Miranda, 2013 [36]**		Cross-Sectional	Participants of the study CAMELIA 2006–2007. DM classification by self-report of a previous diagnosis, hypoglycemic medication and / or FPG ≥126mg/dL.	Immunoassay LabMax 240; Labtest Diagnostica	African-Brazilian adults showed higher HbA1c levels compared with Whites, even after adjusting for potential confounders [6.0% (42 mmol/mol) and 5.8% (40 mmol/mol), respectively]. Blacks had lower levels of income and education and higher frequency of DM and hypertension. HbA1c values for the groups without DM included in our study were made available by the authors.
Whites	190; NI; NI				
Blacks	156; NI; NI				
**Tillin, 2013 [37]**		Cohort	Tri-ethnic population-based cohort from North West London. As contacted with the authors, the analyses presented in the follow-up were based on people without DM.	Ion-Exchange HPLC Tosoh A1C G8; Tosoh Corporation	HbA1c levels were statistically higher in South Asian and African-Caribbean individuals compared with European Whites persons [6.1% (43 mmol/mol), 6.0% (42 mmol/mol) and 5.8% (40 mmol/mol), respectively]. After multivariate analysis, the ethnic differences in HbA1c values remained unchanged.
European Whites	582; 76.9; NI				
African-Caribbean	150; 51.3; NI				
South Asians	341; 82.9; NI				
**Shipman, 2015 [38]**		Cross-Sectional	Demographic and laboratory data from non-diabetic South Asian (Indian, Pakistani and Bangladeshi) and White patients living in the United Kingdom were collected following anonymization.	Ion-Exchange HPLC Tosoh A1C G7; Tosoh Corporation	South Asian patients presented higher HbA1c levels, but lower fasting glucose concentrations than White patients after adjustment for hematological, biochemical and demographic factors [5.9% (41 mmol/mol) and 5.8% (40 mmol/mol), respectively].
Whites	711; 42.8; 57.5				
South Asian	237; 41.8; 47.9				
**Aviles-Santa, 2016 [39]**		Cross-Sectional	Individuals without self-reported diabetes from six Hispanic/Latino heritage groups, enrolled from 2008 to 2011 in the Hispanic Community Health Study/Study of Latinos, and non-Hispanic white adults enrolled during the 2007–2012 cycles of the National Health and Nutrition and Examination Survey.	Ion-Exchange HPLC Tosoh A1C G7, Tosoh Corporation	HbA1c differs among Hispanics/Latinos of diverse heritage groups and between non-Hispanic whites and Hispanics/Latinos after adjustment for glycemia and other covariates.
Whites	NI; NI; NI				
Hispanics/Latinos	NI; NI; NI				
**Carson, 2016 [40]**		Cross-Sectional	Participants of the CARDIA study, data from the year 20 examination (2005–2006). Diabetes was defined as a self-report of a physician diagnosis of diabetes or use of insulin or oral hypoglycemic medications.	Ion-Exchange HPLC Tosoh A1C G7; Tosoh Corporation	African-American patients without diagnosed diabetes had higher mean levels of HbA1c than Whites after multivariable adjustment. The racial differences observed for HbA1c persisted after further adjustment for FPG and 2hG and were of similar magnitude.
Whites	1,445; 48.3; 45.7				
African-Americans	1,100; 41.3; 44.7				

HbA1c, glycated hemoglobin; NI, not informed.

### Quality assessment

The evaluation of the studies quality is shown in Table 2. Of these 12 studies, 10 were classified as being of good quality [19, 20, 22, 24, 25, 34, 35, 38–40] and three met all the quality criteria [20, 35, 38]. Two studies with moderate quality presented insufficient data for criteria selection, study design and statistical analysis. The authors did not describe mean and SD for HbA1c [36, 37], therefore it was necessary to contact them for that information. In addition, one of the works was a symposium poster presentation, without enough data in the abstract.

**Table 2:** Adapted Newcastle-Ottawa quality assessment scale for the studies included in the meta-analysis.

Author (Ref #)	Year	Population Studied	Selection	Study design	Ethnicity	Statistical Analysis	Total Score ^a^
**Burden [34]**	**1999**	**	*	**	**	**	**9**
**Likhari [35]**	**2010**	**	**	**	**	**	**10**
**Kehl [22]**	**2010**	**	*	*	**	**	**8**
**Selvin [24]**	**2013**	**	*	*	**	**	**8**
**Champ-Jumbo [19]**	**2012**	**	**	**	**	**	**10**
**Mostafa [20]**	**2012**	*	**	**	**	**	**9**
**Bower [25]**	**2013**	**	*	*	**	**	**8**
**Miranda [36]**	**2013**	**	*	*	**	*	**7**
**Tilin [37]**	**2013**	*	*	*	*	*	**5**
**Shipman [38]**	**2015**	**	**	**	**	**	**10**
**Avilés-Santa [39]**	**2016**	**	**	**	**	*	**9**
**Carson [40]**	**2016**	**	*	**	*	**	**8**

^a^ The total score ranges from zero to 10 stars, the highest quality studies are awarded a maximum of 10 stars.

The subgroup comparing Black and White persons included a large number of individuals and the majority of the studies presented good quality. It consisted of African Americans, British African Caribbeans and Black Brazilians [19, 22, 24, 25, 36, 37, 40]. The five studies in the subgroup of Asians and Whites were carried out in the United Kingdom and included individuals of South Asian origin, mainly from India and Pakistan [20, 34, 35, 37, 38]. One study had moderate quality [37] due to being a congress communication with insufficient information. Three studies were included in the subgroup that compared Latinos and Whites persons, from United States [22, 25, 39]. The absence of heterogeneity and the good quality of the studies were strong points of this sub-meta-analysis, although two presented research questions slightly different from our own meta-analysis [22, 25].

### Meta-analysis

**Fig 2:**
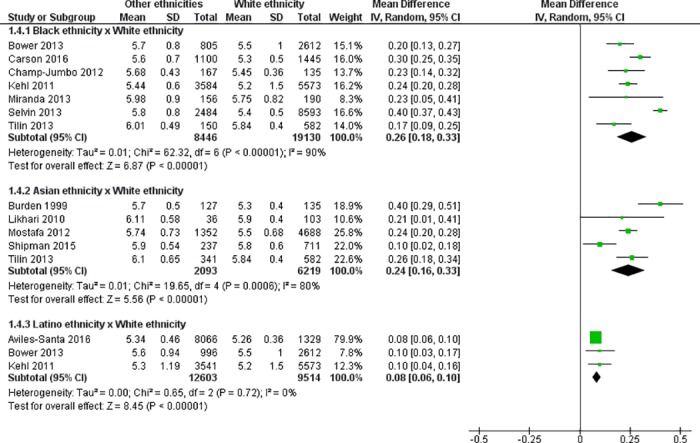
Forest plot diagram of the effect of ethnicity on HbA1c (%) levels in non-diabetic persons; CI = confidence interval; SD = standard deviation.

All studies included in this review had compared HbA1c values in White persons with HbA1c values in Blacks, Asians and/or Latinos. Subgroup analyses were performed based on each ethnicity compared with the control group (White persons). Some studies have been included in more than one subgroup, depending on the ethnic groups analysed (Fig 2).

Seven studies were included in the meta-analysis for Blacks and Whites [19, 22, 24, 25, 36, 37, 40], totalling 27,576 subjects in this subgroup. Blacks had significantly higher HbA1c values than Whites. The difference was of 0.26% [2.8 mmol/mol; 95% CI 0.18 to 0.33 (2.0 to 3.6), p <0.001]; and a high heterogeneity among studies was observed (I^2^ = 90%, p for heterogeneity <0.001). After the sensitivity analysis, by re-running the meta-analysis removing a study at a time, only when excluding the study by Selvin et al [24] the heterogeneity decreased, and we found a similar significant difference [0.24% (2.6 mmol/mol); 95% CI 0.22 to 0.27 (2.4 to 3.0), p <0.001; I^2^ = 49%, p for heterogeneity = 0.08]. When excluding the studies by Selvin et al [24] and by Carson et al [40] together there was no evidence of heterogeneity among studies (I^2^ = 0%, p for heterogeneity = 0.64). We also investigated the effect of HbA1c methodology in the heterogeneity by performing an analysis excluding the study by Miranda et al [36], which was the only study that used immunoassay method. There was no change in the heterogeneity, although we observed a little increase in the difference of HbA1c values between Blacks and Whites (0.30% [3.3 mmol/mol; 95% CI 0.28 to 0.33 (3.1 to 3.6), p <0.001; I^2^ = 92%, p for heterogeneity <0.001) when only studies that measured HbA1c by HPLC were considered. However, after carefully evaluating all studies, we were not able to explain the reasons why they individually contributed to the increase in heterogeneity and they were not excluded from the primary meta-analysis. The visual analysis of funnel plot did not demonstrate publication bias.

In the subgroup comparing Asian and White individuals, 5 studies were included [20, 34, 35, 37, 38], totalling 8,312 individuals. Asians had significant higher HbA1c values than Whites and the difference was of 0.24% [2.6 mmol/mol; 95% CI 0.16 to 0.33 (1.7 to 3.6), p <0.001]. A high heterogeneity among studies was also observed (I^2^ = 80%, p for heterogeneity = 0.0006). After the sensitivity analysis, only when removing the study by Shipman et al [38] there was a decrease in the heterogeneity. The difference in HbA1c levels remained similar however a moderate heterogeneity among studies was observed [0.28% (3.1 mmol/mol); 95% CI 0.21 to 0.35 (2.3 to 3.8), p <0.001; I^2^ = 59%, p for heterogeneity = 0.06]. We were not able to explain the reasons why this study individually contributed to the increase in heterogeneity and it was not excluded from the primary meta-analysis. To investigate the contribution of HbA1c methodology, we re-ran the meta-analysis without the study by Burden et al [34], which used an immunoassay to measure HbA1c, and we found very similar results (0.22% [2.4 mmol/mol; 95% CI 0.19 to 0.25 (2.1 to 2.7), p <0.001; I^2^ = 70%, p for heterogeneity <0.001). The funnel plot showed no significant asymmetry, by visual analysis.

Three studies were included in the subgroup that compared the HbA1c values between Latino and White persons [22, 25, 39], totalling 22,117 individuals. Latinos had higher HbA1c values than Whites, with a low but significant difference and no evidence of heterogeneity among studies [0.08% (0.9 mmol/mol); IC 95% 0.06 to 0.10 (0.7 to 1.1); p <0.001; I^2^ = 0%; p for heterogeneity = 0.72]. In this subgroup all studies used HPLC methodology.

## Discussion

In this systematic review, we have evaluated the effect of ethnicity on HbA1c values in individuals without DM. The absolute HbA1c values were significantly higher in Black (0.26%), Asian (0.24%) and Latino (0.08%) persons when compared to Whites. As far as we know, this is the first systematic review with meta-analysis that compared the effect of ethnicity on HbA1c levels in individuals without DM in whom HbA1c excursions are small.

In 2 previous meta-analysis that evaluated differences in glycaemic control among ethnicities in patients with DM, HbA1c mean value was around 8.0% (64 mmol/mol) for White patients [12, 41]. Relative increments of 8.1% and 6.2% in HbA1c values were estimated for African-Americans and Latinos, respectively, in these studies. In our meta-analysis, HbA1c mean value for White persons was around 5.5% (37 mmol/mol) and we found positive relative differences of 4.7%, 4.4% and 1.5% for Blacks, Asians and Latinos, respectively. Taking into account the results by Kirk et al [12,14] and the results of this meta-analysis, we can assume that other factors beyond blood glucose levels, glycaemic control, the access to health care or the quality of diabetes care, may account for a portion of the variations in HbA1c levels for these ethnic groups. In our review, since all studies apparently evaluated healthy persons, with glucose levels below the cut-off point for DM, we may presume that the differences found in HbA1c values between Blacks, Asians and Latinos and White individuals are probably independent of DM status and/or other factors related to health care. However, it is not possible to affirm that all variables that could influence for glycaemia in these subjects have been properly evaluated in each study. In the literature, differences in HbA1c values were also observed in individuals with pre-diabetes. Blacks, Latinos, American Indians, and Asians presented higher HbA1c values than Whites [14, 42].

The ethnicity did not modify the association between HbA1c and the risk for cardiovascular disease and final-stage renal disease, and prevalent retinopathy in non-diabetic individuals supporting the same interpretation of HbA1c for the prognosis of diabetic complications among all populations [43]. However, differences in the relationship between HbA1c and mean self-monitored blood glucose were found across the different ethnic groups with DM [44]. Recently, a sub-analysis of the Action to Control Cardiovascular Risk in Diabetes (ACCORD) glycaemia trial showed that patients who failed to reach the HbA1c target in the standard treatment group after 1-year of study protocol were more likely to be Black, have severe hypoglycaemia and be on insulin therapy [45].

The physiological mechanisms underlying these differences, at any glucose concentrations, remain unknown. Some biological factors have been suggested as possible causes, such as variations in the glycation gap (defined as the difference between the measured HbA1c and the HbA1c value predicted from glycated serum proteins/fructosamine), differences in erythrocytes survival, variances in haemoglobin glycation, heterogeneity in the glucose concentration gradient across the erythrocyte membranes and differences in the passage of glucose mediated by GLUT1 transporter into the erythrocyte [46–48]. Therefore, one can assume that Black, Asian and Latino populations could possibly present specific physiological characteristics that differentiate them from the White population.

Genetic studies have also been conducted to explore the causes for differences in HbA1c levels among persons of similar blood glucose values. A study conducted in a cohort of nondiabetic twins concluded that 66% of the glycation gap is heritable and the environment influences the other 31% [49]. Another study observed that the contribution of genetic ancestry to HbA1c values seems to represent only a very small portion of the higher HbA1c levels in African-Americans and that socioeconomic and other metabolic factors represent the main causes for that disparity [50]. More recently, fructosamine 3-kinase (FN3K), an enzyme that is related to protein deglycation, may be associated to HbA1c levels [51]. It is involved in the removal of fructosamines, the final products from Amadori rearrangement, and in vitro experiments showed that when erythrocytes were incubated in the presence of glucose and a competitive inhibitor of FN3K, there was an increase about twofold in the rate of accumulation of HbA1c [51]. Moreover, studies have reported that genetic variants may be responsible for increasing or reducing its enzymatic activity and consequently affecting HbA1c levels [52, 53]. However, yet there are no studies comparing the activity or presence of variants of FN3K among different ethnicities and its relationship with HbA1c levels.

It is known that HbA1c levels are lower in Whites when compared to Blacks and other ethnic populations and there is an ongoing debate about what these differences mean and how they impact on the diagnosis and monitoring of people with DM [53–56]. In this meta-analysis we provided information on the amount of these differences, expressed as HbA1c absolute values, in individuals without DM. Whether different therapeutic targets and diagnostic cut-off points for DM should be used among ethnic groups is still unclear [54, 55]. A proper evaluation of the glycemic impact on HbA1c levels in different ethnicities could resolve the issue. However, until now no published study in this field has adequately addressed this question [54]. Despite this, the individualization of glycaemic goals has been highlighted in the recent years and personalized approaches for medical care, based on patient clinical characteristics, are recommended [55, 57].

This study has strengths and limitations. An important high point of this research is that it is the first study to compare the effect of ethnicity on the HbA1c levels in individuals without DM, and based on a comprehensive literature search without any language restriction. Furthermore, we only included studies in which HbA1c was measured by standardized methods and in most studies included, the quality was good. The major limitation of our meta-analysis is that most studies did not provide quantitative data for FPG and/or 2-h plasma glucose. Moreover, no data on postprandial glycaemia were available; therefore we cannot affirm that the differences of HbA1c levels were not at least partially due to differences in glycaemic factors. Some studies have classified the individuals as without DM by self-assessment and not by glucose-based tests. Consequently, we could not perform a meta-regression to explore the blood glucose values as a source of the heterogeneity. In addition, we were unable to measure how much the presence of pre-diabetic individuals has influenced the results of HbA1c in our analysis. Finally, other factors such as exercise, diet, and other related lifestyle behaviors may affect HbA1c levels, leading to potential for residual confounding, and the bias could have been differential by race/ethnicity.

In conclusion, this meta-analysis shows that, in individuals without DM, HbA1c values are higher in Blacks, Asians and Latinos when compared to White persons. Although small, these disparities might have impact on the use of a sole HbA1c cut-off point to diagnose DM in all ethnic populations. Until now, if these differences are clinically relevant is still unknown. Moreover, we have to better understand the mechanisms involved in the HbA1c variability among ethnic groups to improve its clinical applicability.

## Supporting information

S1 Checklist MOOSE checklist. (PDF)

S2 Checklist PRISMA checklist. (PDF)

S1 Appendix Complete search strategy for MEDLINE and EMBASE. (PDF)

S2 Appendix Adapted Newcastle-Ottawa quality assessment scale to evaluate study quality of the studies included in the meta-analysis. (PDF)

## Abbreviations

DM: Diabetes mellitus
FPG: Fasting plasma glucose
FN3K: Fructosamine 3—kinase
IFCC: International Federation for Clinical Chemistry
NGSP: National Glycohemoglobin Standardization Program

## References

[1] American Diabetes Association. Diagnosis and Classification of Diabetes. Diabetes Care. 2016; 39 [Suppl. 1]: S13–S22 26696675 10.2337/dc16-S005

[2] DCCT—The Diabetes Control and Complications Trial Research Group. The effect of intensive treatment of diabetes on the development and progression of long-term complications in insulin-dependent diabetes mellitus. N Engl J Med. 1993; 329:977–86 10.1056/NEJM199309303291401 8366922

[3] U.K. Prospective Diabetes Study (UKPDS) Group: Intensive blood-glucose control with sulphonylureas or insulin compared with conventional treatment and risk of complications in patients with type 2 diabetes (UKPDS 33). Lancet. 1998;352:837–51 9742976

[4] Riddle MC, Ambrosius WT, Brillon DJ, Buse JB, Byington RP, Cohen RM, et al for the Action to Control Cardiovascular Risk in Diabetes Investigators. Epidemiologic relationships between A1C and all-cause mortality during a median 3.4-year follow-up of glycemic treatment in the ACCORD trial. Diabetes Care. 2010;33:983–90 10.2337/dc09-1278 20427682 PMC2858202

[5] World Health Organization (WHO).Use of glycated haemoglobin (HbA1c) in the diagnosis of diabetes mellitus. 2011 Available at: http://www.who.int/diabetes/publications/report-hba1c_2011.pdf. 26158184

[6] Kramer CK, Araneta MR, Barrett-Connor E. A1C and diabetes diagnosis: The Rancho Bernardo Study. Diabetes Care. 2010;33(1):101–3 10.2337/dc09-1366 19837792 PMC2797952

[7] Cavagnolli G, Comerlato J, Comerlato CB, Renz PB, Gross JL, Camargo JL. HbA1c measurement for the diagnosis of diabetes: is it enough? Diabet Med. 2011;28(1):31–5 21210540 10.1111/j.1464-5491.2010.03159.x

[8] Hare MJ, Shaw JE, Zimmet PZ. Current controversies in the use of haemoglobin. J Intern Med. 2012;271(3):227–36 10.1111/j.1365-2796.2012.02513.x 22333004

[9] Sacks DB, Bruns DE, Goldstein DE, Maclaren NK, McDonald JM, Parrott M. Guidelines and recommendations for laboratory analysis in the diagnosis and management of diabetes mellitus. Clin Chem. 2002; 48(3): 436–72 11861436

[10] Cavagnolli G, Pimentel AL, Correa Freitas PA, Gross JL, Camargo JL. Factors affecting A1C in non-diabetic individuals: Review and meta-analysis. Clin Chim Acta. 2015;445:107–14 10.1016/j.cca.2015.03.024 25818244

[11] Silva JF, Pimentel AL, Camargo JL. Effect of iron deficiency anaemia on HbA1c levels is dependent on the degree of anaemia. Clin Biochem. 2016;49(1):117–20 26365695 10.1016/j.clinbiochem.2015.09.004

[12] Kirk JK, D'Agostino RB Jr, Bell RA, Passmore LV, Bonds DE, Karter AJ, et al Disparities in HbA1c levels between African-American and non-Hispanic White adults with diabetes: a meta-analysis. Diabetes Care. 2016; 29:2130–6 10.2337/dc05-1973 PMC3557948 16936167

[13] Herman WH, Cohen RM. Racial and ethnic differences in the relationship between HbA1c and blood glucose: implications for the diagnosis of diabetes. J Clin Endocrinol Metab. 2012; 97(4):1067–72 10.1210/jc.2011-1894 22238408 PMC3319188

[14] Herman WH, Ma Y, Uwaifo G, Haffner S, Kahn SE, Horton ES, et al Diabetes Prevention Program Research Group: Differences in A1C by race and ethnicity among patients with impaired glucose tolerance in the Diabetes Prevention Program. Diabetes Care. 2007;30:2453–7 10.2337/dc06-2003 17536077 PMC2373980

[15] De Rekeneire N, Rooks RN, Simonsick EM, Shorr RI, Kuller LH, Schwartz AV, et al Racial differences in glycemic control in a well-functioning older diabetic population: Findings from the Health, Aging and Body Composition study. Diabetes Care. 2007; 26:1986–92 10.2337/diacare.26.7.1986 12832300

[16] Herman WH, Dungan KM, Wolffenbuttel BHR, Buse JB, Fahrbach JL, Jiang H, et al Racial and Ethnic Differences in Mean Plasma Glucose, Hemoglobin A(1c), and 1,5-Anhydroglucitol in Over 2000 Patients with Type 2 Diabetes. J Clin Endocrinol Metab. 2009;94:1689–94 10.1210/jc.2008-1940 19276235

[17] Stratton IM, Adler AI, Neil HA, Matthews DR, Manley SE, Cull CA, et al Association of glycaemia with macrovascular and microvascular complications of type 2 diabetes (UKPDS 35): prospective observational study. BMJ. 2000;321:405–12 10938048 10.1136/bmj.321.7258.405 PMC27454

[18] Selvin E, Steffes MW, Ballantyne CM, Hoogeveen RC, Coresh J, Brancati FL. Racial Differences in Glycemic Markers: A Cross-sectional Analysis of Community-Based Data. Ann Intern Med. 2011;154(5):303–9 10.7326/0003-4819-154-5-201103010-00004 21357907 PMC3131743

[19] Chapp-Jumbo E, Edeoga C, Wan J, Dagogo-Jack S. Pathobiology of Prediabetes in a Biracial Cohort (POP-ABC) Research Group. Ethnic disparity in hemoglobin A1c levels among normoglycemic offspring of parents with type 2 diabetes mellitus. Endocr Pract. 2012;18(3):356–62 10.4158/EP11245.OR 22138077

[20] Mostafa SA, Davies MJ, Webb DR, Srinivasan BT, Gray LJ, Khunti K. Independent Effect of Ethnicity on Glycemia in South Asians and White Europeans. Diabetes Care. 2012;35(8):1746–8 10.2337/dc11-2079 22699291 PMC3402276

[21] Mostafa SA, Davies MJ, Webb DR, Gray LJ, Srinivasan BT, Jarvis J, et al The potential impact of using glycated haemoglobin as the preferred diagnostic tool for detecting Type 2 diabetes mellitus. Diabet Med. 2010;27(7):762–9 10.1111/j.1464-5491.2010.03015.x 20636956

[22] Kehl KG, Findeisen HM, Fardo DW, Bruemmer D, Mannino DM, Sanderson WT. Race-ethnicity as an effect modifier of the association between HbA1c and mortality in US adults without diagnosed diabetes. Eur J Endocrinol. 2011; 165:275–81 10.1530/EJE-11-0171 21622476

[23] Ziemer DC, Kolm P, Weintraub WS, Vaccarino V, Rhee MK, Twombly JG, et al Glucose-independent, Black-White differences in hemoglobin A1c levels: A cross-sectional analysis of 2 studies. Ann Intern Med. 2010;152(12):770–7 10.7326/0003-4819-152-12-201006150-00004 20547905

[24] Selvin E, Rawlings AM, Bergenstal RM, Coresh J, Brancati FL. No racial differences in the association of glycated hemoglobin with kidney disease and cardiovascular outcomes. Diabetes Care. 2013;36(10):2995–3001 10.2337/dc12-2715 23723353 PMC3781554

[25] Bower JK, Brancati FL, Selvin E. No Ethnic Differences in the Association of Glycated Hemoglobin With Retinopathy The National Health and Nutrition Examination Survey 2005–2008. Diabetes Care. 2013;36(3):569–73 10.2337/dc12-0404 23069841 PMC3579340

[26] Christensen DL, Witte DR, Kaduka L, Jørgensen ME, Borch-Johnsen K, Mohan V, et al Moving to an A1C-based diagnosis of diabetes has a different impact on prevalence in different ethnic groups. Diabetes Care. 2010;33(3):580–2 10.2337/dc09-1843 20009099 PMC2827511

[27] Higgins JPT, Green S (editors). Cochrane Handbook for Systematic Reviews of Interventions Version 5.1.0 [updated March 2011]. The Cochrane Collaboration, 2011 Available from www.cochrane-handbook.org.

[28] Stroup DF, Berlin JA, Morton SC, Olkin I, Williamson GD, Rennie D, et al Meta-analysis of observational studies in epidemiology: a proposal for reporting. Meta-analysis Of Observational Studies in Epidemiology (MOOSE) group. JAMA. 2000;283:2008–12 10789670 10.1001/jama.283.15.2008

[29] Hanas R, John G. International HbA₁(c) Consensus Committee. 2010 Consensus statement on the worldwide standardization of the hemoglobin A(1c) measurement. Diabetes Res Clin Pract. 2010;90(2):228–30 10.1016/j.diabres.2010.05.011 20598392

[30] Little RR, Rohlfing CL, Wiedmeyer HM, Myers GL, Sacks DB, Goldstein DE, et al The National Glycohemoglobin Standardization Program: A five-year report progress. Clin Chem. 2001;47:1985–92 11673367

[31] Christenson RH, Snyder SR, Shaw CS, Derzon JH, Black RS, Mass D, et al Laboratory medicine best practices: systematic evidence review and evaluation methods for quality improvement. Clin Chem. 2011;57(6):816–25 10.1373/clinchem.2010.157131 21515742

[32] Wells GA, Shea B, O'Connell D, Peterson J, Welch V, Losos M, et al. The Newcastle-Ottawa Scale (NOS) for assessing the quality of nonrandomized studies in metanalysis. Available at: http://www.ohri.ca/programs/clinical_epidemiology/oxford.asp.

[33] Egger M, Smith GD, Schneider M, Minder C. Bias in metaanalysis detected by a simple, graphical test. BMJ. 1997;315:629–634 9310563 10.1136/bmj.315.7109.629 PMC2127453

[34] Burden ML, Basi M, Burden AC. HbA(1c) local reference ranges: Effects of age, sex and ethnicity. Practical Diabetes International. 1999;16(7): 211–4

[35] Likhari T, Gama R. Ethnic differences in glycated haemoglobin between White subjects and those of South Asian origin with normal glucose tolerance. J Clin Pathol. 2010;63:278–80 10.1136/jcp.2009.065821 20203232

[36] de Miranda VA, Cruz Filho RA, de Oliveira TS, Moscavitch SD, Kang HC, Miranda Chagas SV, et al Racial differences in HbA1c: A cross-sectional analysis of a Brazilian public primary care population. Prim Care Diabetes. 2013;7(2):135–41 10.1016/j.pcd.2013.01.007 23485345

[37] Tillin T, Hughes AD, Godsland IF, Tuson C, Forouhi NG, Sattar N, et al Do risk factors measured 20 years previously predict and explain ethnic differences in HbA1c among non-diabetic British South Asians, African Caribbeans and Europeans? Diabet Med. 2013;30 [suppl s1]:76(Abstract)

[38] Shipman KE, Jawad M, Sullivan KM, Ford C, Gama R. Ethnic/racial determinants of glycemic markers in a UK sample. Acta Diabetol 2015;52(4):687–92 10.1007/s00592-014-0703-y 25559352

[39] Avilés-Santa ML, Hsu LL, Arredondo M, Menke A, Werner E, Thyagarajan B, et al Differences in Hemoglobin A1c Between Hispanics/Latinos and Non-Hispanic Whites: An Analysis of the Hispanic Community Health Study/Study of Latinos and the 2007-2012 National Health and Nutrition Examination Survey. Diabetes Care 2016;39(6):1010–7 10.2337/dc15-2579 27208330 PMC5317242

[40] Carson AP, Muntner P, Selvin E, Carnethon MR, Li X, Gross MD, et al Do glycemic marker levels vary by race? Differing results from a cross-sectional analysis of individuals with and without diagnosed diabetes. BMJ Open Diabetes Res Care 2016 10;4(1):e000213 10.1136/bmjdrc-2016-000213 27335652 PMC4908883

[41] Kirk JK, Passmore LV, Bell RA, Narayan KM, D'Agostino RB Jr, Arcury TA, et al Disparities in A1C levels between Hispanic and non-Hispanic White adults with diabetes: a meta-analysis. Diabetes Care. 2008;31(2):240–6 10.2337/dc07-0382 17977939

[42] Likhari T, Gama R. Glycaemia-independent ethnic differences in HbA(1c) in subjects with impaired glucose tolerance. Diabet Med. 2009;26(10):1068–9 10.1111/j.1464-5491.2009.02803.x 19900241

[43] Parrinello CM, Sharrett AR, Maruthur NM, Bergenstal RM, Grams ME, Coresh J, et al Racial Differences in and Prognostic Value of Biomarkers of Hyperglycemia. Diabetes Care. 2016;39:4589–95 10.2337/dc15-1360 PMC4806772 26681712

[44] Wolffenbuttel BHR, Herman WH, Gross JL, Dharmalingam M, Jiang HH, Hardin DS. Ethnic Differences in Glycemic Markers in Patients With Type 2 Diabetes. Diabetes Care. 2013;36(10): 2931–6 10.2337/dc12-2711 23757434 PMC3781497

[45] Drake TC, Hsu FC, Hire D, Chen SH, Cohen RM, McDuffie R, et al Factors associated with failure to achieve a glycated haemoglobin target of <8.0% in the Action to Control Cardiovascular Risk in Diabetes (ACCORD) trial. Diabetes Obes Metab. 2016;18(1):92–5 10.1111/dom.12569 26435375 PMC6241305

[46] Gould BJ, Davie SJ, Yudkin JS. Investigation of the mechanism underlying the variability of glycated haemoglobin in non-diabetic subjects not related to glycaemia. Clin Chim Acta. 1997;260(1):49–64 9101100 10.1016/s0009-8981(96)06508-4

[47] Cohen RM, Holmes YR, Chenier TC, Joiner CH. Discordance between HbA(1c) and fructosamine—Evidence for a glycosylation gap and its relation to diabetic nephropathy. Diabetes Care. 2003;26(1):163–7 12502674 10.2337/diacare.26.1.163

[48] Khera PK, Joiner CH, Carruthers A, Lindsell CJ, Smith EP, Franco RS, et al Evidence for interindividual heterogeneity in the glucose gradient across the human red blood cell membrane and its relationship to hemoglobin glycation. Diabetes. 2008;57 (9): 2445–52 10.2337/db07-1820 18591386 PMC2518496

[49] Cohen RM, Snieder H, Lindsell CJ, Beyan H, Hawa MI, Blinko S, et al Evidence for independent heritability of the glycation gap, (glycosylation gap) fraction of HbA(1c) in nondiabetic twins. Diabetes Care. 2006;29(8):1739–43 10.2337/dc06-0286 16873773

[50] Maruthur NM, Kao WH, Clark JM, Brancati FL, Cheng CY, Pankow JS, et al Does genetic ancestry explain higher values of glycated hemoglobin in African Americans? Diabetes. 2011;60(9): 2434–8 10.2337/db11-0319 21788574 PMC3161314

[51] Van Schaftingen E, Collard F, Wiame E, Veiga-da-Cunha M. Enzymatic repair of Amadori products. Amino Acids. 2012;42(4): 1143–50 10.1007/s00726-010-0780-3 20967558

[52] Avemaria F, Carrera P, Lapolla A, Sartore G, Chilelli NC, Paleari R, et al Possible role of frutosamine 3-kinase genotyping for the management of diabetic patients. Clin Chem Lab Med. 2015;53(9):1315–20 10.1515/cclm-2015-0207 26352355

[53] Mohás M, Kisfali P, Baricza E, Mérei A, Maász A, Cseh J, et al A polymorphism within the fructosamine-3-kinase gene is associated with HbA1c Levels and the onset of type 2 diabetes mellitus. Exp Clin Endocrinol Diabetes. 2010 3;118(3):209–12 10.1055/s-0029-1238319 19834870

[54] Herman WH. Are There Clinical Implications of Racial Differences in HbA1c? Yes, to Not Consider Can Do Great Harm! Diabetes Care. 2016; 39(8):1458–61. 10.2337/dc15-2686 27457636 PMC4955925

[55] Sacks DB. Hemoglobin A1c and Race: Should Therapeutic Targets and Diagnostic Cutoffs Differ among Racial Groups? Clin Chem. 2016; 62(9):1199–201. 10.1373/clinchem.2016.255166 27386851

[56] Selvin E. Are There Clinical Implications of Racial Differences in HbA1c? A Difference, to Be a Difference, Must Make a Difference. Diabetes Care. 2016; 39(8):1462–7. 10.2337/dc16-0042 27457637 PMC4955930

[57] Ismail-Beigi F, Moghissi E, Tiktin M, Hirsch IB, Inzucchi SE, Genuth S. Individualizing glycemic targets in type 2 diabetes mellitus: implications of recent clinical trials. Ann Intern Med.2011;154(8):554–9 10.7326/0003-4819-154-8-201104190-00007 21502652

