# Supplementary material for: Effect of ethnicity on HbA1c levels in individuals without diabetes: Systematic review and meta-analysis
Source: PLoS One. 2017 Feb 13;12(2):e0171315. doi: 10.1371/journal.pone.0171315 (PMC5305058; doi:10.1371/journal.pone.0171315)
Supplement: S1 Checklist — (PDF) [file pone.0171315.s001.pdf]

MOOSE Checklist

From: Stroup DF, Berlin JA, Morton SC, et al (2000) Meta-analysis of observational studies in epidemiology: A proposal for reporting. JAMA 283:2008–2012. doi:10.1001/jama.283.15.2008.

	Reported on page	Comments
**Reporting of background should include**		
Problem definition	4;5	
Hypothesis statement	5	
Description of study outcome(s)	5;6	
Type of exposure or intervention used	5	
Type of study designs used	6	
Study population	6	
**Reporting of search strategy should include**		
Qualifications of searchers (e.g. librarians and investigators)	7	
Search strategy, including time period used in the synthesis and key words	5;6	S1 Appendix (Supplemental Data)
Effort to include all available studies, including contact with authors	5;6	
Databases and registries searched	5	
Search software used, name and version, including special features used (e.g. explosion)	5	
Use of hand searching (e.g. reference lists of obtained articles)	5	
List of citations located and those excluded, including justification	8, 9	Also Figure 1, Table 1
Method of addressing articles published in languages other than English	5	No papers in languages other than English were eligible for inclusion in the steps of full text analysis.
Method of handling abstracts and unpublished studies	7	
Description of any contact with authors	6 and 7	
**Reporting of methods should include**		
Description of relevance or appropriateness of studies assembled for assessing the hypothesis to be tested	6	
Rationale for the selection and coding of data (e.g. sound clinical principles or convenience)	6 and 7	
Documentation of how data were classified and coded (e.g. multiple raters, blinding and interrater reliability)	7	
Assessment of confounding (e.g. comparability of cases and controls in studies where appropriate)	8	
Assessment of study quality, including blinding of quality assessors, stratification or regression on possible predictors of study results	7 and 8	
Assessment of heterogeneity	8	
Description of statistical methods (e.g. complete description	8	

of fixed or random effects models, justification of whether the chosen models account for predictors of study results, dose-response models, or cumulative meta-analysis) in sufficient detail to be replicated		
Provision of appropriate tables and graphics	10-13, 15,30;31	Tables 1 and 2; Figures 1 and 2
**Reporting of results should include**		
Graphic summarizing individual study estimates and overall estimate	31	Figure 2
Table giving descriptive information for each study included	10-13	Table 1
Results of sensitivity testing (e.g. subgroup analysis)	16,17	
Indication of statistical uncertainty of findings	16,17,31	Figure 2
**Reporting of discussion should include**		
Quantitative assessment of bias (e.g. publication bias)	17,18	
Justification for exclusion (e.g. exclusion of non-English language citations)	17,18,19	No papers in languages other than English were eligible for inclusion in the steps of full text analysis.
Assessment of quality of included studies	15	Table 2
**Reporting of conclusions should include**		
Consideration of alternative explanations for observed results	18,19	
Generalization of the conclusions (i.e. appropriate for the data presented and within the domain of the literature review)	19,20	
Guidelines for future research	20	
Disclosure of funding source	21	
