# Supplementary material for: Effect of ethnicity on HbA1c levels in individuals without diabetes: Systematic review and meta-analysis
Source: PLoS One. 2017 Feb 13;12(2):e0171315. doi: 10.1371/journal.pone.0171315 (PMC5305058; doi:10.1371/journal.pone.0171315)
Supplement: S1 Appendix — (PDF) [file pone.0171315.s003.pdf]

Appendix (Supplementary Data):

The complete search strategy for MEDLINE.

((((((((Hemoglobins[Title/Abstract]) OR Hemoglobin A, Glycosylated[Title/Abstract]) OR Hb A1c[Title/Abstract]) OR Glycosylated Hemoglobin A[Title/Abstract]) OR Hemoglobin, Glycosylated[Title/Abstract]) OR Glycosylated Hemoglobin[Title/Abstract]) OR Glycated Hemoglobins[Title/Abstract]) OR Hemoglobins, Glycated[Title/Abstract])) AND (((((Ethnic Groups[Title/Abstract]) OR African Continental Ancestry Group[Title/Abstract]) OR Oceanic Ancestry Group[Title/Abstract]) OR European Continental Ancestry Group[Title/Abstract]) OR Asian Continental Ancestry Group[Title/Abstract])

The complete search strategy for EMBASE.

'hemoglobin'/exp AND a1c AND [embase]/lim OR glycosylated AND 'hemoglobin'/exp AND [embase]/lim AND 'ethnicity'/exp AND [embase]/lim OR ethnic AND racial AND groups AND [embase]/lim OR ethnic AND group AND [embase]/lim OR ethnic, AND racial AND religious AND groups AND [embase]/lim
