# Supplementary material for: Effect of ethnicity on HbA1c levels in individuals without diabetes: Systematic review and meta-analysis
Source: PLoS One. 2017 Feb 13;12(2):e0171315. doi: 10.1371/journal.pone.0171315 (PMC5305058; doi:10.1371/journal.pone.0171315)
Supplement: S2 Appendix — (PDF) [file pone.0171315.s004.pdf]

S2 Appendix. Adapted Newcastle-Ottawa quality assessment scale to evaluate study quality of the studies included in the meta-analysis.

TOTAL SCORE: 10 stars

1) **Population Studied: Total of Stars = 2**
   a. Full description of the population studied: age, sex, clinical origin? If Yes, **two stars**
   b. Description without one or more items missing. If Yes, **one star**

2) **Selection of Participants: Total of Stars = 2**
   a. Participants are identified without DM by FG and/or OGTT? If Yes, **two stars**
   b. Exclusion of DM was done by interview, previous clinical history and/or familiar history? If Yes, **one star**

3) **Study Design: Total of Stars = 2**
   a. The study design was in accordance with the purpose of research question under study. If Yes**, two star**
   b. The study was designed for another purpose, but provided sufficient data for our analysis. If yes, **one star**

4) **Interfering factor: Total of Stars = 2**
   a. The factor under investigation was well described (for example: levels of uremia; levels of total Hb or Hb variant type or other diagnostic criteria). If Yes, **two stars**
   b. The factor under investigation was partially described by interview, previous clinical history and/or familiar history. If Yes, **one star**

5) **Statistical Analysis: Total of Stars = 2**
   a. Patients without DM had their A1C results clearly described (mean, SD and/or SEM). If Yes, **two stars**
   b. Results for A1C for patients without DM were obtained after contact with the authors. If Yes, **one star**
